# An LC-MS/MS Method for Simultaneous Determination of the Toxic and Active Components of* Cortex Periplocae* in Rat Plasma and Application to a Pharmacokinetic Study

**DOI:** 10.1155/2019/1639619

**Published:** 2019-02-13

**Authors:** Zhen Li, Yang Li, Jin Li, Rui Liu, Jia Hao, Jun He, Yan-xu Chang

**Affiliations:** ^1^Tianjin State Key Laboratory of Modern Chinese Medicine, Tianjin University of Traditional Chinese Medicine, Tianjin 300193, China; ^2^Tianjin Key Laboratory of Phytochemistry and Pharmaceutical Analysis, Tianjin University of Traditional Chinese Medicine, Tianjin 300193, China

## Abstract

A sensitive and simple liquid chromatography-tandem mass spectrometry (LC-MS/MS) method was developed and validated to simultaneously determine the toxic and other active components including isovanillin, scopoletin, periplocin, periplogenin, and periplocymarin after oral administration of* cortex periplocae* extract to rats. Plasma samples were prepared by protein precipitation with methanol. All compounds were separated on a C_18_ column with gradient elution using acetonitrile and formic acid aqueous solution (0.1%,* v/v*) as the mobile phase at a flow rate of 0.3 mL/min. The detection of all compounds was accomplished by multiple-reaction monitoring (MRM) in the positive electrospray ionization mode. The LC-MS/MS method exhibited good linearity for five analytes. The lower limit of quantification (LLOQ) was 0.48 ng/mL for scopoletin, periplogenin, and periplocymarin; 2.4 ng/mL for isovanillin and periplocin. The extraction recoveries of all compounds were more than 90% and the RSDs were below 10%. It was found that the absorption of scopoletin and periplocin was rapid* in vivo* after oral administration of* cortex periplocae* extract. Furthermore, periplocymarin possessed abundant plasma exposure. The results demonstrated that the validated method was efficiently applied for the pharmacokinetic studies of isovanillin, scopoletin, periplocin, periplogenin, and periplocymarin after oral administration of* cortex periplocae *extract.

## 1. Introduction

Traditional Chinese Medicines (TCMs) play the vital roles in the prevention and treatment of diseases and are used in clinical practice for centuries in China [1]. In view of the commonly used TCMs, some of them are toxic such as* Spanish fly *[2],* Radix Aconiti *[3],* Strychnos nux-vomica L. *[4], etc. In spite of their toxicity, these TCMs still were widely used in clinic due to their special pharmacological effects. It is worth noting that the clinical adverse reaction of the toxic TCMs limits their applications in clinical therapy. Thus, the safety of the toxic TCMs becomes increasingly important. Pharmacokinetic study was helpful for evaluating the safety by analyzing the absorption, distribution, metabolism, and excretion of drugs* in vivo* [1, 5]. Thus, it is essential to perform the pharmacokinetic study of the toxic and/or other active components in the toxic TCMs for achieving the best therapeutic effects and reducing the toxicity of TCMs [6, 7].

*Cortex periplocae*, called ‘xiangjiapi' as a toxic traditional Chinese medicine (TCM) [8, 9], is the root bark of* Periploca sepium *Bge. (family: Asclepiadaceae) [10]. It has widely been used for relieving rheumatic conditions and slaking dropsy as well as strengthening the bone and the musculature clinically for many years [11, 12]. At present, many constituents in c*ortex periplocae*, such as cardiac glycosides, sterides, oligosaccharides, pregnane glycosides, coumarins, flavonoids, and triterpenoides, have been identified [13, 14]. It is reported that cardiac glycosides such as periplocin, periplogenin, and periplocymarin were not only the toxic components of c*ortex periplocae *[8], but also the active components exerted cardiac effect and anticancer activity [15–17]. Isovanillin has the antispasmodic [18] and antidiarrheal activity [19]. Moreover, some previous findings suggested that scopoletin possessed anti-inflammatory, antithyroid, antioxidative, and antihyperglycemic activity, etc. [20–22]. Therefore, it is necessary to clarify the pharmacokinetic fates of these toxic and active components for evaluating the safety of c*ortex periplocae*.

There were several analytical methods for determining the toxic or active components in herbal medicine or in biological fluids, such as the simultaneous determination one or multiple compounds as well as their optimization of extraction conditions [23–25], the pharmacokinetics and/or tissue distribution studies of the three toxic components [26–29], and the pharmacokinetic study on scopoletin in Caulis* Erycibes *[30]. There was also a report about the absorption parameters of periplocin* in vitro* by HPLC [31]. However, there is no report for simultaneous determination the toxic and active components in biological fluids after an oral administration of* cortex periplocae* extract. In the present study, the three toxic and active components (periplocin, periplogenin, and periplocymarin) and other two active components (isovanillin and scopoletin) in rat plasma were simultaneously quantified by liquid chromatography-tandem mass spectrometry (LC-MS/MS). The newly established LC-MS/MS method was successfully applied to the pharmacokinetic study.

## 2. Material and Methods

### 2.1. Chemicals, Reagents, and Materials

**Figure 1:**
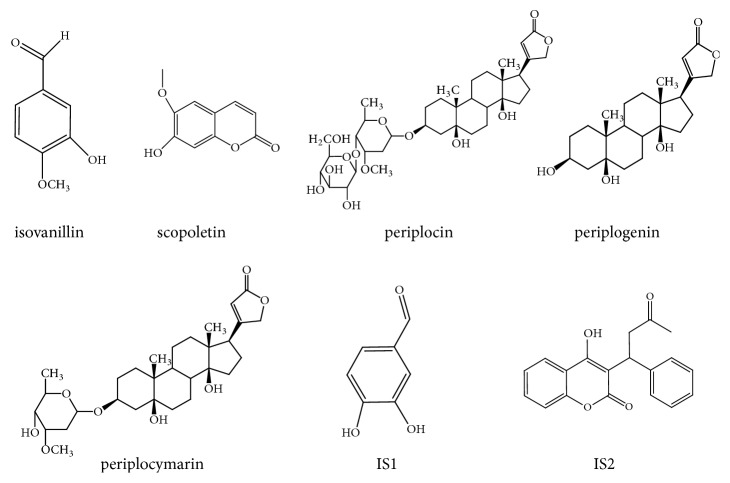
Chemical structures of five analytes and two ISs (IS1: protocatechuic aldehyde; IS2: warfarin).

Acetonitrile and methanol from Merck (Darmstadt, Germany), as well as formic acid (ROE, Newark, USA), were HPLC grade. Deionized water was prepared by a Milli-Q system (Millipore, MA, USA). Periplocin (purity: 99.18%), periplocymarin (purity: 99.93%), periplogenin (purity: 99.81%), and scopoletin (purity ≥98%) were supplied from Chengdu Dest Biotechnology Co., Ltd. (Chengdu, China). Isovanillin (purity: 98%) was obtained from Tianjin Vientiane Hengyuan Technology Co., Ltd. (Tianjin, China). Protocatechuic aldehyde (purity: 99%) and warfarin (purity: 92.3%) were provided from National Institute for the Control of Pharmaceutical and Biological Products (Beijing, China). The chemical structures of all compounds are shown in Figure 1.* Cortex periplocae *was purchased from Shanxi province and the voucher specimen was deposited at Tianjin University of Traditional Chinese Medicine, Tianjin, China.

### 2.2. Apparatus and Analytical Conditions

The LC-MS/MS equipment consisted of an Agilent 1200 LC system (a binary pump, a vacuum degasser unit, a Hip-ALS autosampler) and an API 3200 triple quadruple mass spectrometer (Concord, Ontario, Canada) equipped with an electrospray ionization (ESI) source.

Chromatographic separation was achieved on an analytical column (Eclipse plus C_18_, 4.6 mm × 100 mm, 1.8 *μ*m) with a security guard C_18_ column (2.1 mm × 12.5 mm, 5 *μ*m) (Agilent, USA) maintained at 20°C. The mobile phase consisted of acetonitrile (A) and formic acid aqueous solution (0.1%,* v/v*) (B) using a gradient elution as follows: 30-70% A at 0-20 min; 70-75% A at 20-25 min, at a flow rate of 0.3 mL/min, and the re-equilibration time of gradient elution was 5 min. The injection volume was 15 *μ*L. The compounds were monitored in multiple-reaction monitoring (MRM) mode with the positive electrospray ionization. The ion transitions were* m/z *153.1→93.0 for isovanillin, 193.1→133.1 for scopoletin, 719.6→719.4 for periplocin, 391.3→355.5 for periplogenin, 535.3→355.3 for periplocymarin, 139.4→65.2 for protocatechuic aldehyde (IS1), and 309.3→163.2 for warfarin (IS2). The optimal responses of all analytes were obtained when ion spray voltage was 4500 V with temperature at 550°C and curtain gas at 10 psi. In addition, GS1 and GS2 were 40 psi and 40 psi for isovanillin, scopoletin,, periplocin, periplogenin, periplocymarin, 50 psi for IS1, and 50 psi for IS2.

### 2.3. The Preparation of Cortex Periplocae Extract

*Cortex periplocae *(2.0 kg) was extracted with eight-fold volumes of 95% alcohol and six-fold volumes of 60% alcohol under heat reflux method for 2 h, successively. C*ortex periplocae* extract was obtained when the combined extract was concentrated to dryness under reduced pressure. The extraction yield was 15%.

### 2.4. Preparation of Calibration Standard and Quality Control Solutions

Appropriate amount of each compound was weighed and dissolved in methanol to achieve a concentration of 1.0 mg/mL as the primary stock solution. The mixture of IS (internal standard) stock solution was prepared by diluting the stock solution with methanol and the final concentrations were 500 ng/mL and 20 ng/mL for protocatechuic aldehyde and warfarin, respectively. For the calibration standard solutions, appropriate volume of each analyte stock solution was mixed together to obtain the desired mixed solution and the mixture was diluted serially with methanol. The quality control (QC) solutions were prepared following the calibration standard solutions preparation method. All solutions were stored at 4°C.

### 2.5. Preparation of Plasma Samples and Quality Control (QC) Samples

To a 100 *μ*L aliquot of plasma sample in a centrifuge tube, 10 *μ*L of IS and 300 *μ*L methanol were successively added. The mixture was vortexed for 1 min and centrifuged for 10 min at 14000 rpm. The supernatant was transferred into another centrifuge tube and then centrifuged at 14000 rpm for 10 min. QC samples were prepared by adding 10 *μ*L QC solutions to a centrifuge tube and 10 *μ*L of IS, the bank rat plasma (100 *μ*L) and methanol (300 *μ*L) were successively added, and then the mixture followed the same method as that for the plasma samples to achieve the concentrations of 1.44, 24, and 240 ng/mL for scopoletin, periplogenin, and periplocymarin; 7.2, 120, 1200 ng/mL for isovanillin and periplocin, respectively.

### 2.6. Method Validation

#### 2.6.1. Specificity

The specificity was evaluated by comparing the chromatogram of blank plasma sample from six different batches rats with those of the blank plasma spiked with analytes (LLOQ) and IS, as well as real plasma samples obtained from rat after administration of* cortex periplocae* extract at a dose of 18.9 g/kg.

#### 2.6.2. Calibration Curve and Sensitivity

Calibration samples were prepared by spiking 10 *μ*L calibration standard solutions into a centrifuge tube and then followed by the same method as that for preparation of QC samples to achieve the concentrations in the linearity range of 0.48-300 ng/mL (0.48, 0.8, 2.4, 4, 12, 20, 60, 100, and 300 ng/mL) for scopoletin, periplogenin, and periplocymarin; 2.4-1500 ng/mL (2.4, 4, 12, 20, 60, 100, 300, 500, and 1500 ng/mL) for isovanillin and periplocin. The calibration curves of all analytes were calculated by the peak-area ratios of analytes to IS which were plotted against the nominal concentrations using a 1/X weighting. The lower limit of quantification (LLOQ) that could be accurately and precisely determined was the lowest concentration in the calibration curve.

#### 2.6.3. Precision and Accuracy

The intra-day and inter-day precision and accuracy were estimated by analyzing six replicate QC samples on one and three consecutive days, respectively. The relative standard deviation (RSD) was used to assess the precision and the percent ratios of the calculated concentrations to the nominal concentrations were defined as the accuracy.

#### 2.6.4. The Recovery and Matrix Effect

The recovery and matrix effect were determined by QC samples at three levels. The recovery was assayed by comparing the determined peak areas of analytes in processed plasma samples with those of post-processed spiked samples. The matrix effect was evaluated by normalized matrix factors, the peak area ratio of analytes, and corresponding IS in post-processed spiked samples to that in non-processed samples. The recovery of IS was evaluated at a final concentration.

#### 2.6.5. Stability

The QC samples (n = 6) at three concentrations (low, medium, and high) were also used to assess the stabilities of all analytes. The QC samples under autosampler condition for 24 h were determined to evaluate the autosampler 24 h stability. For the freeze/thaw cycle stability, the QC samples stored at -80°C were subjected to three freeze to thaw (at room temperature) cycles. QC samples stored at room temperature for 24 h and -80°C for 1 month were used to evaluate the short-term stability and the long-term stability. The mixed analytes solution (100 ng/mL), which was obtained by diluting the stock solution stored for two weeks at 4°C, was used to evaluate the stability of stock solution. The stability of analytes working solution was also assessed by storing solutions 2 weeks at 4°C. The stabilities of stock and working solutions of ISs were determined by the same procedure as that for the analytes.

#### 2.6.6. Carryover

Carryover was determined by successively injecting LLOQ sample and ULOQ sample (blank plasma sample spiked with the highest concentration at the calibration curve) and three blank plasma samples. It was considered that the carryover did not affect the LC-MS/MS assay when the analyte and IS responses in the blank plasma samples were lower than 20% of the LLOQ response and 5% of IS working solutions, respectively.

### 2.7. Applications to Pharmacokinetic Study

The pharmacokinetic study was conducted in accordance with the Guidelines for the Care and Use of Laboratory Animals and approved by the Animal Ethics Committee of Tianjin University of Traditional Chinese Medicine. Male Sprague–Dawley rats (260-300 g) housed to a cage were fasted for 12 h and allowed free access to water prior to the experiment. Appropriate amount of* cortex periplocae* extract was suspended in 0.5% carboxymethyl cellulose sodium salt aqueous solution (CMC-Na) and the volume of oral administration was 1 mL/100g. According to the clinical dose, the rats were given* cortex periplocae* extract at an oral dose of 18.9 g/kg. The rats were anesthetized with ethyl ether before each withdrawal. The blood samples (about 250 *μ*L) were collected into centrifuge tubes with heparin sodium from the suborbital vein at 0.033, 0.083, 0.167, 0.25, 0.5, 1, 2, 4, 6, 8, 12, 24, 36, and 48 h after dose. For this repeat bleeds at short intervals, fluid replacement (appropriate volume of physiological saline) was required. The blood samples were immediately centrifuged 10 min at 7000 rpm. The obtained plasma was transferred into another centrifuge tube and stored at -80°C for analysis.

### 2.8. Data Analysis

For the maximum drug concentration in plasma (C_max_) and the time to reach maximum drug concentration (T_max_), the values could be directly obtained. The other key parameters such as the area under the plasma concentration–time curve (AUC), elimination half-life (t_1/2_), and mean residence time (MRT) were calculated with DAS (Drug and Statistics 1.0, Medical College of Wannan, China) using non-compartmental model.

## 3. Results and Discussion

### 3.1. Optimization of LC-MS/MS Method

In order to separate all compounds well, the composition and proportion of the mobile phase were investigated. The results indicated that acetonitrile and formic acid aqueous solution (0.1%,* v/v*) with a finial gradient elution provided the best separation of all compounds. The flow rate was 0.3 mL/min. For the mass conditions, the ion intensities of analytes and IS in the negative-ion mode were not high as that in the positive-ion mode. Moreover, source parameters were also optimized to enhance the sensitivity and achieve better responses.

### 3.2. Optimization of Sample Preparation

An efficient sample preparation process could achieve a desired recovery and eliminate the interference from endogenous components from the matrix. The precipitating protein with acetonitrile/methanol methods and liquid-liquid extraction with ethyl acetate methods were investigated to extract all analytes from samples, respectively. The results indicated that protein precipitation with methanol could obtain satisfactory extraction recovery. Finally, the plasma sample processed by the protein precipitation with methanol was directly centrifuged two times at 14000 rpm for 10 min. Subsequently, 15 *μ*L solutions were injected into the LC-MS/MS system for analysis.

### 3.3. Assaying the Dosage of Oral Administration of Four Compounds

The contents of isovanillin, scopoletin, periplocin, periplogenin, and periplocymarin in* cortex periplocae* extract were determined by LC-MS/MS. The results showed that the dosage of isovanillin, scopoletin, periplocin, periplogenin, and periplocymarin was 32.76 mg/kg, 7.09 mg/kg, 28.89 mg/kg, 2.21 mg/kg, and 1.34 mg/kg, respectively.

### 3.4. Method Validation

#### 3.4.1. Specificity

**Figure 2:**
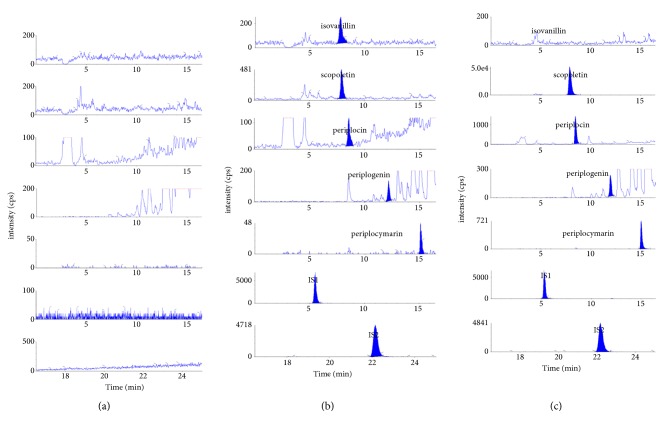
Typical chromatograms of specificity: (a) blank rat plasma, (b) the blank plasma spiked with five analytes and two ISs (IS1: protocatechuic aldehyde; IS2: warfarin), and (c) plasma sample.

The typical chromatograms of all compounds are presented in Figure 2. The retention time of isovanillin, scopoletin, periplocin, periplogenin, periplocymarin, IS1 (protocatechuic aldehyde), and IS2 (warfarin) was 7.5 min, 7.72 min, 8.28 min, 12.16 min, 15.06 min, 5.49 min, and 22.12 min, respectively. As shown in Figure 2, there were no interferences from endogenous substances in the plasma during the determination.

#### 3.4.2. Calibration Curve and Sensitivity

The calibration curve equations were as follows: y = 0.00728 x - 0.000117 for isovanillin, y = 0.265 x + 0.0211 for scopoletin, y = 0.00366 x + 0.072 for periplocin, y = 0.0424 x + 0.00747 for periplogenin, and y = 0.0168 x - 0.000106 for periplocymarin. All analytes showed good linearities as indicated by the correlation coefficient (r) more than 0.99. The LLOQ was 0.48 ng/mL for scopoletin, periplogenin, and periplocymarin; 2.4 ng/mL for isovanillin and periplocin, which indicated that the sensitivity of the established method was excellent.

#### 3.4.3. Precision and Accuracy

The values of intra-day and inter-day precision and accuracy are summarized in Table 1. For all analytes, the RSDs of the intra-day and inter-day were less than 13% and the accuracies ranged from 92.6% to 115%. The results indicated that the method was precise and accurate for determining the concentration of all analytes in rat plasma over the linearity ranges.

#### 3.4.4. The Recovery and Matrix Effect

The results of recovery and matrix effect are presented in Table 1. The recoveries of all analytes were more than 90% and RSDs were less than 10%. The matrix effect of all analytes ranged from 0.87 to 1.08 with the RSDs less than 11%. The results indicated that the established protein precipitation with methanol method was efficient to extract all analytes and no significant endogenous substances from plasma were observed to suppress or enhance the ionization of analytes. The recovery of two ISs (protocatechuic aldehyde and warfarin) was 81.6% and 88.6%, respectively.

#### 3.4.5. Stability

The stability data were summarized in Table 2. The results showed that no obvious reduction in plasma were observed, indicating that the analytes in plasma were stable for 24 h in the autosampler and 24 h at room temperature, three freeze/thaw cycles at -80°C and 1 month at -80°C. The working solutions of analytes were also stable as described in Table 2. As for the working solutions of ISs (IS1: protocatechuic aldehyde; IS2: warfarin), the accuracy was 101% and 109% with the RSD of 4.93% and 4.91%, respectively. Furthermore, analytes as well as ISs stock solutions were stable indicated by the data of 86.2%-112% with the RSDs less than 5%.

#### 3.4.6. Carryover

There were no peaks in any of the three blank samples injected into the LC-MS/MS after the injection of an ULOQ sample. It is obviously that the carryover did not affect the determination of analytes and ISs.

### 3.5. Pharmacokinetic Study

**Figure 3:**
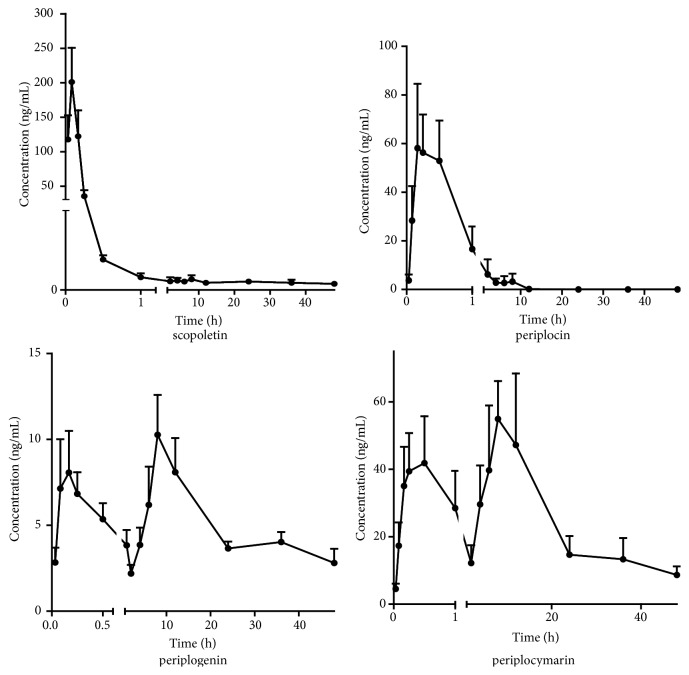
The mean plasma concentration-time profiles of four components after oral administration of* cortex periplocae* extract (n = 6, mean ± SD).

The developed LC-MS/MS method was successfully employed to determine the concentration of isovanillin, scopoletin, periplocin, periplogenin, and periplocymarin in rat plasma after oral administration of* cortex periplocae* extract at a dose of 18.9 g/kg. It was notable that isovanillin was not detected in rat plasma after oral administration, which could be attributed to the following reasons: its concentration in rat plasma was lower than LLOQ or it was not absorbed after oral administration. In addition, the mean plasma concentration-time profiles were displayed in Figure 3. It was obvious that the non-compartmental model was most suitable to describe the pharmacokinetic profiles of analytes. The major pharmacokinetic parameters of scopoletin, periplocin, periplogenin, and periplocymarin were presented in Table 3. The results indicated that scopoletin and periplocin achieved the maximum plasma concentration in a short time, particularly scopoletin with T_max_ of 0.10 ± 0.03 h. According to the results, periplogenin was faster than periplocymarin reaching the maximum plasma concentration which was consistent with previous report [26]. As shown in Figure 3, periplocymarin and periplogenin appeared with double peaks and the second peak was higher than the first peak; one of the reasons for such peak appearance patterns was that periplocin in rat was metabolized to periplocymarin and periplogenin. Compared to the previous paper [26], periplocin could be determined and the concentration would be lower than LLOQ after oral administration for 12 h; the proper reason was that other constituents from* cortex periplocae* extract promoted the absorption of periplocin, and C_max_ is only 9 times higher for periplocymarin (125.96 ± 96.42 ng/mL) than for periplogenin (14.53 ± 7.75 ng/mL), which attributed to the effect of other constituents from* cortex periplocae* extract. The concentration of scopoletin in plasma decreased rapidly.

C_max_ was 177.83 ± 133.85 ng/mL for scopoletin, which indicated that the concentration of scopoletin in plasma was high while periplogenin obtained the low plasma concentration. In addition, periplocymarin (AUC_(0-48 h)_ = 1689.73 ± 1174.92 ng h/mL) possessed abundant plasma exposure. t_1/2_ of periplocin and scopoletin was 1.21 ± 1.11 h and 0.19 ± 0.08 h, which showed their elimination was also quick.

### 3.6. Method Comparison with Existing Reports

A few studies on periplocin, periplogenin, and periplocymarin in biological fluids [20–22] have been reported. However, these analytical methods did not simultaneously involve the toxic components and other active components. Additionally, the pharmacokinetic characteristics of scopoletin after oral administration of* cortex periplocae *extract have not been published despite the pharmacokinetics in Caulis* Erycibes* have been carried out [31]. In this paper, the other active components (isovanillin and scopoletin) besides three toxic components were also determined by LC-MS/MS. Meanwhile, the sample preparation which was accomplished by a direct precipitation protein with methanol was simpler and faster than those of previous methods.

## 4. Conclusion

A developed and validated LC-MS/MS method was successfully applied to simultaneous determination of the toxic and other active components (isovanillin, scopoletin, periplocin, periplogenin, and periplocymarin) in rat plasma after oral administration of c*ortex periplocae* extract and evaluating their pharmacokinetic characteristics. The results suggested that the absorption and elimination of scopoletin and periplocin was rapid. In addition, periplogenin and periplocymarin appeared with double peaks. These pharmacokinetic results could provide more information of analytes for the development of c*ortex periplocae* in clinical practice. The pharmacokinetic characteristics were useful to evaluate the clinical efficacy as well as safety of analytes from c*ortex periplocae* extract and design rational dosage regimens of* cortex periplocae*. Furthermore, these pharmacokinetic results would be of great help to understand its pharmacokinetics and promote the development of* cortex periplocae* in clinical practice.

## Figures and Tables

**Table 1:** The accuracy, precision, recoveries, and matrix effects.

Compounds	Concentration (ng/mL)	Intra-day		inter-day		Recovery		Matrix effect	
		Accuracy (%)	RSD (%)	Accuracy (%)	RSD (%)	Accuracy (%)	RSD (%)	Normalized matrix factor	RSD (%)
isovanillin	7.2	106	3.58	99.6	5.18	97.1	3.56	1.08	4.45
	120	111	2.19	102	13.0	97.1	2.31	1.06	2.46
	1200	108	2.55	105	7.59	104	1.73	1.04	1.79
scopoletin	1.44	108	2.92	107	1.88	102	6.68	1.00	3.32
	24	109	2.77	106	2.13	103	5.39	0.98	1.91
	240	96.4	4.51	95.2	2.33	98.1	2.81	1.06	2.49
periplocin	7.2	111	4.26	115	3.82	100	7.79	1.01	10.7
	120	109	4.02	104	3.21	97.7	6.67	0.98	4.18
	1200	101	6.34	110	6.07	106	3.40	0.99	2.06
periplogenin	1.44	106	3.69	108	1.32	90.9	5.60	0.98	9.71
	24	110	2.71	107	4.21	98.0	6.23	0.97	1.68
	240	95.4	4.03	97.9	4.11	102	2.19	0.91	3.18
periplocymarin	1.44	101	7.64	107	8.09	103	9.75	0.87	7.79
	24	106	3.70	102	7.25	99.5	5.94	0.96	3.96
	240	101	3.20	95.3	8.76	104	1.97	0.89	1.96
IS1	300	-	-	-	-	81.6	2.12	-	-
IS2	20	-	-	-	-	88.6	6.18	-	-

**Table 2:** Stability of analytes.

Compounds	Concentration (ng/mL)	Working solution		Autosampler for 24 hours		Room temperature for 24 h		Freeze thaw cycles		-80°C for 1 month	
		Accuracy (%)	RSD (%)	Accuracy (%)	RSD (%)	Accuracy (%)	RSD (%)	Accuracy (%)	RSD (%)	Accuracy (%)	RSD (%)
isovanillin	7.2	104	4.27	100	2.31	107	7.01	101	1.97	109	7.54
	120	96.1	4.79	112	1.88	106	2.57	98.6	1.80	100	5.58
	1200	85.0	3.77	114	3.19	103	2.13	107	2.32	99.4	7.46
scopoletin	1.44	103	1.29	104	4.19	96.8	6.45	104	2.36	108	8.17
	24	109	4.24	105	3.30	115	4.27	110	2.74	108	5.42
	240	97.3	4.90	97.1	1.74	103	1.01	100	2.72	96.4	5.79
periplocin	7.2	103	1.92	113	1.65	111	8.33	93.7	6.72	115	8.62
	120	98.9	4.58	109	4.02	116	1.80	114	9.17	115	9.91
	1200	94.4	4.41	113	1.60	96.4	4.23	113	2.53	92.9	6.10
periplogenin	1.44	111	1.80	108	3.12	92.6	4.17	116	4.69	109	7.47
	24	99.4	3.71	110	3.69	115	5.21	112	3.25	108	3.45
	240	81.5	2.68	104	4.19	101	2.04	92.8	2.68	98.7	5.48
periplocymarin	1.44	110	3.13	101	4.71	107	5.14	113	5.91	106	7.77
	24	95.1	4.41	108	5.38	102	5.85	113	1.74	102	3.27
	240	84.2	1.73	102	4.27	98.5	1.99	103	2.28	102	5.81

**Table 3:** Pharmacokinetic parameters of the analytes (n = 6, mean ± SD).

parameters	scopoletin	periplocin	periplogenin	periplocymarin
T_max_ (h)	0.10 ± 0.03	0.31 ± 0.16	5.78 ± 3.56	5.49 ± 4.34
Cmax (ng/mL)	177.8 ± 133.85	91.37 ± 52.95	14.53 ± 7.75	125.96 ± 96.42
AUC(0-tn) (ng h/mL)	103.00 ± 76.36	82.98 ± 58.62	206.57 ± 130.58	1689.73 ± 1174.92
AUC(0-∞) (ng h/mL)	205.81 ± 152.57	105.05 ± 70.88	446.29 ± 174.38	2258.69 ± 1284.45
t_1/2_ (h)	0.19 ± 0.08	1.08 ± 0.98	22.91 ± 17.30	30.18 ± 11.83
MRT(0-tn) (h)	11.81 ± 6.60	1.21 ± 1.11	19.07 ± 3.64	17.15 ± 2.48

## Data Availability

The data used to support the findings of this study are included within the article.
